# Empathy Mediates the Relations between Working Memory and Perpetration of Intimate Partner Violence and Aggression

**DOI:** 10.3390/bs10030063

**Published:** 2020-03-05

**Authors:** Donald A. Godfrey, Caitlin M. Kehoe, Adriana Bastardas-Albero, Julia C. Babcock

**Affiliations:** Department of Psychology, University of Houston, Houston, TX 77204, USA; ckehoe@cougarnet.uh.edu (C.M.K.); abastardas-albero@uh.edu (A.B.-A.); jbabcock@Central.uh.edu (J.C.B.)

**Keywords:** executive functioning, working memory, interpersonal violence, empathy

## Abstract

Deficits in executive functioning have been associated with aggressive and violent behavior toward intimate partners. However, it is unclear what specific mechanisms are being affected by cognitive deficits that increase an individual’s tendency to become aggressive. The current study examined empathy as a mediating factor between deficits in working memory and perpetration of intimate partner aggression and violence. Men in heterosexual relationships (*N =* 49) were administered a measure of visual-spatial working memory, and questionnaire measures of head injury and empathy. During a second session, men participated in a conflict discussion with their female partner that was coded for aggressive behavior. Female partners also reported on men’s physical and psychological abuse. Working memory was positively related to cognitive and affective empathy, and negatively related to men’s physical abuse perpetration and observed aggression during the conflict discussion. The effects of working memory on observed aggression during the conflict were fully mediated by cognitive and affective empathy. Additionally, the effects of working memory on reported physical IPV frequency were fully mediated by affective empathy. Deficits in working memory may decrease men’s ability to use empathetic processes, resulting in increased aggression and violence toward their intimate partners. Clinically, incorporating empathy training in battering intervention programs may be helpful, especially among men with deficits in cognitive functioning.

## 1. Introduction

Intimate Partner Violence (IPV) is a significant public health concern within the United States today. An estimated 43 million women and 38 million men have been impacted throughout their lifetime by IPV [1]. It is estimated that among women in the United States, approximately 1300 deaths and 2 million injuries occur annually as a result of violence perpetrated by an intimate partner [2]. Additionally, victims of IPV are highly susceptible to substance abuse, depression, and chronic illness [3]. Current treatments aimed at reducing IPV perpetration are not highly effective [4]. Accordingly, there is a significant need to increase theoretical knowledge of the associated factors of IPV perpetration to improve prevention and treatment strategies.

Researchers examining associated factors of IPV have consistently found that IPV perpetrators have significantly higher rates of severe head injury and traumatic brain injury compared to men who have not perpetrated IPV [5,6,7]. These rates are stable across methods of reporting, including self-reported loss of consciousness and physician diagnosis of a concussion [8]. The presence of head injury among IPV perpetrators has been associated with decreased cognitive functioning. For example, within a sample of convicted IPV perpetrators, those who had experienced a head injury performed worse on neuropsychological tests and subtests of IQ tests after their injury, controlling for age and IQ prior to the injury [9]. Accordingly, researchers have begun to employ a multidimensional conceptualization of perpetration of violence against partners to include neuropsychological mechanisms [10,11]. Perpetrators of IPV have been found to perform significantly worse than nonviolent controls on various cognitive domains, including executive function, learning, memory, attention, and verbal functioning [5,8,10,12,13,14,15]. Although increased head injury and decreased executive functioning appear be associated with IPV perpetration, it is unclear why. One possible explanation is certain cognitive deficits affect empathy, which facilitates the expression of violence and aggression.

Head injury and neuropsychological functioning appears to be associated with social cognitive domains related to empathy. For example, individuals who have experienced a traumatic brain injury (TBI) often exhibit deficits in social cognition and cognitive empathy [15,16]. Furthermore, patients with TBI and brain atrophy perform worse on measures of empathy compared to healthy controls [17,18]. This relation to brain morphology and empathetic processes is not surprising, as theoretically, empathy requires the ability to decode visual and verbal information from individuals in their environment, and then hold that collective information long enough to develop an understanding of that person’s experience. Indeed, deficits in these processes, particularly working memory, have been associated with decreases in empathetic functioning [19,20,21].

The construct of empathy is generally separated into cognitive empathy, which refers to one’s ability to understand the psychological point of view of another, and affective empathy, which refers to one’s orientation toward others’ emotions [22]. Researchers have suggested that these two factors of empathy have bi-directional causation in social cognitive processes [23]. That is, an individual’s ability to understand another’s feelings improves their ability to understand the other’s perspective, and a greater understanding of one’s perspective increases their understanding of the other’s feelings. Researchers have found that, for non-violent couples, empathy plays a significant role in navigating interpersonal situations and is positively correlated with relationship quality [24]. Among violent men, empathy is negatively associated with psychopathology and violent behavior toward intimate partners [25,26]. Empathy is thought to have an inhibitory effect on a person’s tendency to aggress due to the cognitive dissonance that is experienced [27], which, in turn, serves to reduce the risk of violence [28]. Thus, empathic functioning appears to be highly influenced by neuropsychological functioning, particularly in working memory, as empathy relies on the ability of an individual to hold and organize information of another adequately enough to build a mental representation of their cognitive and emotional experience. Accordingly, empathy may serve as a mediating factor between cognitive functioning and aggression among intimate partners.

The current study examined the relations between empathic traits, visual-spatial working memory, concussion history, and IPV. The first aim within this study was to examine the effects of concussion history on working memory, empathy, partner reported violence, and observed aggression during a conflict discussion with an intimate partner. We hypothesized that individuals who have sustained a concussion would have poorer working memory and lower empathy, as well as greater violence and aggression toward their intimate partner. The second aim of the study was to examine empathy as a mediating factor in the relation between visual-spatial working memory and IPV. Specifically, we hypothesized that poor working memory would be associated with high rates of aggression and violence towards their intimate partners, and that this association would be explained by decreases in cognitive and affective empathy.

## 2. Materials and Methods

Forty-nine heterosexual couples were recruited from a large city in the southwestern United States via flyers and local newspaper ads seeking “couples experiencing conflict.” Couples must have met the following criteria in order to be part of the study: (a) married or living together for at least 6 months, (b) at least 18 years of age, and (c) verbal and written English proficiency. To be classified as IPV, female partners had to report at least two instances of male-to-female IPV within the last year. Women’s IPV perpetration was free to vary and not analyzed in this study. To be classified as non-violent, females had to report that the couples had had zero severe male-to-female violent acts ever and zero minor male-to-female violent acts within the last 5 years. Female partners were advised in advance to refrain from participating in the study if they thought participating would risk their safety. Follow-up phone calls were conducted seven days after participating in the study to make sure there were no unanticipated effects of the study. No woman reported any violence as a result of participating in the study.

### 2.1. Measures

**Corsi Block Tapping Task (CBTT).** The Corsi Block Tapping Task serves as a measure of visuospatial working memory, specifically memory for location and sequence [29]. In this study, the CBTT was modified to an electronic version, using a computer platform. Research has indicated that digital versions of the CBTT demonstrate similar results to those conducted in-person [30]. The participants observed as a square appeared on the screen in isolation at a specific location for one second. Initially, the participant saw two squares which then gradually increased to 12 squares. After the final square appeared, a delay of 0.0001 s occurred. Then, participants were asked to indicate the location of the squares in the order that they were displayed on a grid that appeared on the screen. Participants completed 12 trials in only the forward sequence. Percentile of correct spatial-sequencing scores were operationalized as working memory. Higher percentages indicated increased performance on the CBTT and more effective visual-spatial processing and working memory.

**Intimate partner violence** [31]. The Conflict Tactics Scale 2 (CTS2) is a 78-item questionnaire that assesses intimate partner violence victimization and perpetration within the last year. The CTS2 is broken up into five subscales, including physical assault, physical injury, sexual coercion, psychological abuse, and negotiation. The CTS2 is rated on a 7-point Likert scale, from 0 (*this has never happened)* to 6 (*more than 20 times*). Within this study, women report on the psychological abuse, and physical abuse subscales were used. The CTS2 had alpha value for physical IPV and psychological aggression of 0.80 and 0.79, respectively.

**Observed aggression**. Trained undergraduate research assistants coded displayed aggression using the Specific Affect coding system (SPAFF-16, [32]). SPAFF coding system was used because of its strong reliability and predictive qualities for both violent and non-violent couples during marital interaction tasks [33]. Emotions are measured by analyzing verbal content and tone, body posture, facial expression, and conversational context [34]. As an observational tool, it prevents the biases seen generally in self-reports, including participants’ lack of introspective ability, misinterpretation of questions, and response bias. Research assistants were trained to identify 16 general codes from facial affect, body position and contents of speech. The research assistants entered the onset of each behavior. For these analyses, aggression was calculated as the sum of three affects displayed during the conflict discussion: belligerence, contempt, and domineering. Within the SPAFF coding system, belligerence was described as lifting of the chin and insightful and degrading comments. The inter-rater reliability for belligerence was good, with kappa value of 0.87. Domineering behavior was marked by the lifting of the outer eyebrows, leaning forward, and interrupting and lecturing speech. The inter-rater reliability for the dominance affect was excellent, with a Kappa value of 0.95. Contempt was characterized by the asymmetrical lifting of the outer eyebrow or lip, passive-aggressive or sarcastic remarks, and rolling eyes. The inter-rater reliability for contempt was good with a kappa value of 0.84 [35].

**Empathy [36]**. The Interpersonal Reactivity Index (IRI) was completed by men only during the first session of the study. The IRI is a 28-item self-report measure used to assess empathy. It includes the following four subscales with seven items each: perspective taking, fantasy, empathic concern, and personal distress [36]. The IRI is rated on a 5-point Likert scale, from A (*does not describe me well*) to E (*describes me very well*). For this study, cognitive empathy and affective empathy were operationalized using the “Perspective Taking” and “Empathic Concern” scales, respectively [37]. The IRI demonstrated low to good reliability with an alpha value of 0.81 for cognitive empathy/perspective taking and 0.60 for affective empathy/empathic concern.

### 2.2. Procedures

All procedures were first approved by the University of Houston IRB prior to any data collection. Additionally, collection procedures met ethical standards for conducting research that involved women who potentially have been victims of intimate partner violence set forth by the WHO [38]. Data were collected as part of a larger study examining affective, psychopathology and reactivity of couples with an IPV perpetrator. The study consisted of two sessions. During the first session, men completed a neuropsychological battery, including the Corsi Block Test. Men also completed a self-report on their history of head injury. Men indicated whether or not they had experienced a concussion by responding “*yes*” or “*no*” to the question “*Have you ever had a concussion* (“*seen stars?*” *or* “*had your bell rung*”). In the second session, men returned with their female intimate partner to engage in a conflict discussion. Two topics were chosen from responses on the Knox Problem Inventory. This inventory includes ten different items that couples tend to disagree about with two additional options for couples to add their own topics. Couples then report on a scale of 0–100, with a zero indicating that they do not disagree on the topic, and 100 indicating that they disagree very much. Research assistants identified two items that both participants or at least one participant indicated to be a topic of strong disagreement. Research assistants then led couples through the Play-by-Play interview [39], during which the research assistant presented the two selected items and determined if the topics was suitable for a fifteen-minute conversation based off of the couple’s initial response (i.e., whether the couple quickly dismisses the topic or whether the couple responds to the topics with an affect indicative of distress). Couples then discussed agreed upon problems for 15 min. The conflict discussion was video recorded for later SPAFF coding.

### 2.3. Data Analytic Strategy

Data was assessed for normality by assessing skewness and kurtosis of the study variables (skewness < 2 and kurtosis < 7 [40]). First, we examined differences between those who have suffered a concussion and those who had not across all study variables using a multivariate analysis of variance (MANOVA [41]). Correlations were run between all variables of interest. Then, we conducted four mediation analysis using Hayes Process Macro version 3.3 in SPSS version 26. Within each mediation analysis, we first examined the relation between the predictor variable (working memory) and the mediator variable (affective empathy or cognitive empathy) in a single regression. Second, we ran a multiple regression with both the mediator and the predictor variable estimating the outcome (observed aggression and IPV perpetration). Third, the direct effects of the predictor on the outcome variable were tested without the mediator in a single regression. Lastly, the indirect effect of the predictor on the outcome variable, through the mediator, was estimated using bootstrap confidence intervals sampled at a rate of 10,000 [42].

## 3. Results

### 3.1. Demographics and Descriptive Statistics

Demographic information was self-reported by men of each dyad including age, ethnicity and yearly income. The sample was comprised of male and female individuals between the ages of 18 and 60 years old (*M* = 32.33, *SD* = 9.56, *M* = 29.57, *SD* = 8.79, respectively). Male and female participants reported individual income ranged between $0 and $70,000 (*M=* $22,795, *SD* = $18,788, *M=* $21,284, *SD* = $13,117, respectively). Relationship length within our study ranged from six months to thirty years with an average of 4.59 years (*SD* = 5.15). Additionally, 27 men and 14 women identified as African American, 5 men and 9 women identified as Hispanic, and 15 men and 11 women identified as Caucasian, 3 men and 2 women identified as Asian, and 1 man and 3 women identified as “Other” ethnicity. Within our sample, the majority of men had perpetrated physical IPV (64%) and psychological IPV (88%) within the last year. Men’s physical assault frequency ranged from 0–40 acts in the past year, as reported by their female partners.

All study variables had skewness and kurtosis values within acceptable ranges. Fifteen men (31%) reported suffering at least one concussion. A MANOVA revealed there were no significant differences compared to those with no history of concussion (See Table 1).

Bivariate correlations of the study variable displayed in Table 1 indicate that, as hypothesized, male aggression during the conflict and IPV perpetration was negatively related to both affective empathy and cognitive as well as working memory. Additionally, working memory was positively related to both affective empathy and cognitive empathy. Although there was a trending correlation, psychological aggression rate was not significantly related to working memory or cognitive empathy, but was significantly negatively related to affective empathy. Thus, mediation models estimating psychological aggression were not tested.

### 3.2. Estimating Observed Aggression During Interpersonal Conflict

**Figure 1:**
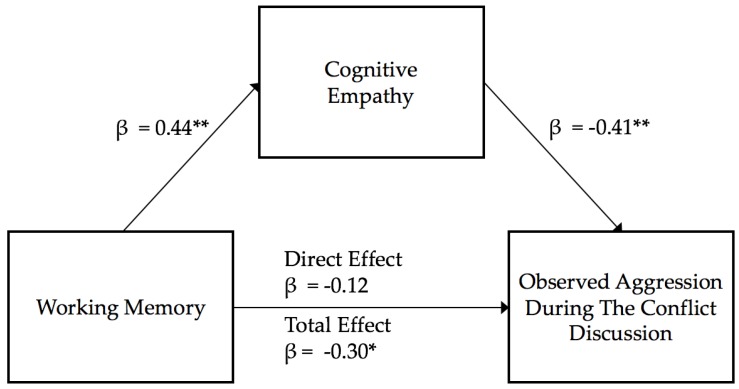
Cognitive empathy mediation model estimating observed aggression. Note: ** *p* < 0.01; * *p* < 0.05.

**Cognitive empathy as a mediator.** In the first regression model of working memory estimating cognitive empathy, working memory estimated a significant amount of the variance in perspective taking (R^2^ = 0.20, *F* (1,47) = 11.51, β = 0.44, *p* = 0.001), as better working memory was associated with higher cognitive empathy. In the second step, the multiple regression of working memory and cognitive empathy explained a significant amount of the variance in male aggression observed during the conflict (R^2^ = 0.23, *F* (2,46) = 6.79, *p* < 0.01), as working memory was not a significant predictor of male aggression (*t* = −0.85, β = −0.12, *p* = 0.40), but cognitive empathy was negatively related to male aggression (*t* = −2.84, β = −0.41, *p* = 0.01). In the third step, testing the direct effects of working memory on male aggression, working memory was significantly negatively related to male observed aggression (R^2^ = 0.09, *F*(1,47) = 4.80, β = −0.30, *p* = 0.03). Visualization of the examined mediation model is presented in Figure 1. The bootstrapped standardized indirect effect of working memory on male aggression, through cognitive empathy was significant (β = −0.18, 95% CI [−0.31, −0.06]), as better working memory was associated with lower male aggression through better cognitive empathy. Thus, cognitive empathy was a mediator between working memory and observed aggression towards the partner.

**Figure 2:**
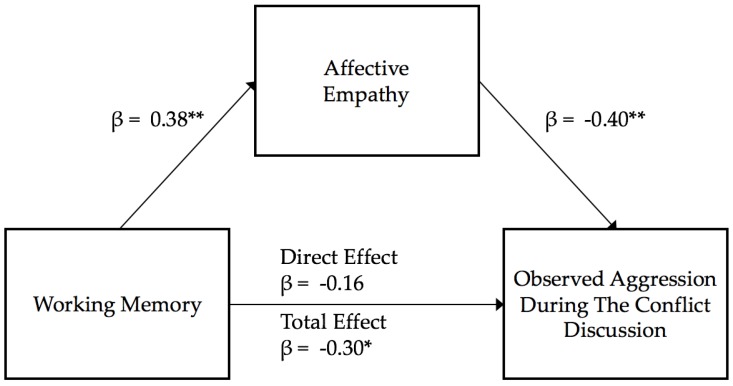
Affective empathy mediation model estimating observed aggression. Note: ** *p* < 0.01; * *p* < 0.05.

**Affective empathy as a mediator.** In the first regression model of working memory estimating affective empathy, working memory estimated a significant amount of the variance in affective empathy (R^2^ = 0.214, *F* (1,47) = 7.72, β = 0.38, *p* < 0.01), as better working memory was associated with better affective empathy. In the second step, the multiple regression of working memory and affective empathy explained a significant amount of the variance in male aggression during the conflict (R^2^ = 0.23, *F* (2,46) = 6.79, *p* < 0.01), as working memory was not a significant predictor of male aggression (*t* = −1.11, β = −0.16, *p* = 0.27). However, affective empathy was negatively related to aggression (*t* = −2.84, β = −0.40, *p* < 0.01). In the third step, testing the direct effects of working memory on male aggression, working memory was significantly negatively related to male observed aggression (R^2^ = 0.09, *F* (1,47) = 4.80, β = −0.30, *p* = 0.03). Visualization of the examined mediation model is presented in Figure 2. The bootstrapped standardized indirect effect of working memory on male aggression, through affective empathy was significant (β = −0.15, 95% CI [−0.319, −0.002]), as better working memory was associated with low male aggression through better affective empathy. That is, affective empathy fully mediated the link between men’s working memory and his aggression observed in the lab.

### 3.3. Estimating Frequency of Male Physical IPV Perpetration

**Figure 3:**
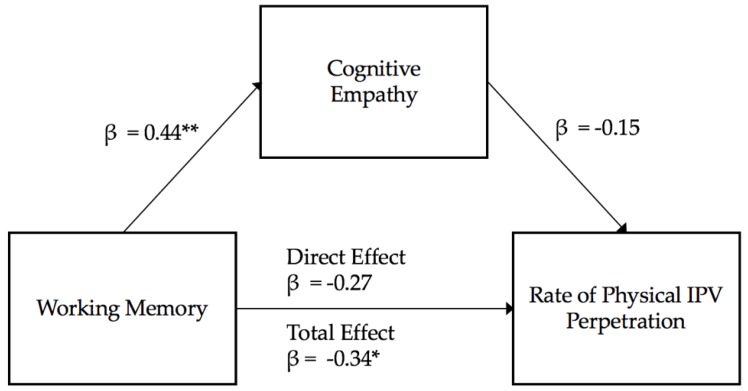
Cognitive Empathy mediation model estimating physical IPV perpetration. Note: ** *p* < 0.01; * *p* < 0.05.

**Cognitive empathy as a mediator.** Working memory and cognitive empathy explained a significant amount of the variance in physical IPV perpetration (R^2^ = 0.23, *F* (2,46) = 6.79, *p* < 0.01). Yet, cognitive empathy was not a significant predictor of women’s reports of his violence (*t* = −0.96, β = −0.15, *p* = 0.34) and working memory tended to predict physical IPV perpetration (*t* = −1.76, β = −0.27, *p* = 0.08). As cognitive empathy was not a significant predictor in the model, indirect mediation effects could not be tested. Visualization of the examined mediation model is presented in Figure 3.

**Figure 4:**
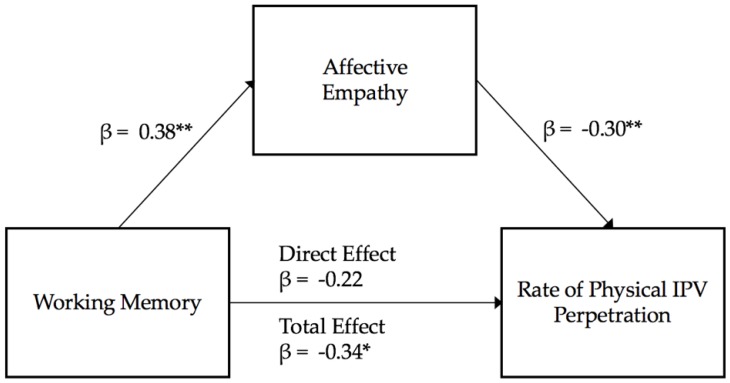
Affective Empathy mediation model estimation physical IPV perpetration. Note: ** *p* < 0.01; * *p* < 0.05.

**Affective empathy as a mediator.** The overall model for affective empathy and working memory was significant in explaining the variance in physical IPV perpetration (R^2^ = 0.19, *F* (2,46) = 5.36, *p* < 0.01). Working memory was not a significant predictor of physical IPV perpetration (*t* = −1.56, β = −0.22, *p* = 0.13), but affective empathy was significantly negatively related to men’s physical IPV perpetration (*t* = −2.08, β = −0.30, *p* = 0.04). In the third step, testing the direct effects of working memory on physical IPV perpetration, working memory was significantly negatively related to male physical IPV perpetration (R^2^ = 0.10, *F* (1,47) = 5.897, β = −0.34, *p* = 0.02). Visualization of the examined mediation model is presented in Figure 4. The bootstrapped standardized indirect effect of working memory on physical IPV perpetration, through affective empathy was significant (β = −0.11, 95% CI [−0.30, −0.001]), as better working memory was associated with decreased physical IPV perpetration through better empathetic concern. As with observed aggression, affective empathy fully mediated the link between men’s working memory and their physical abuse perpetration.

## 4. Discussion

Consistent with current models linking empathy to executive functioning [19], we found that the negative relation between visual-spatial working memory and aggressive behavior and violence frequency towards intimate partners was fully mediated by empathy.

Mediation analysis revealed that visual-spatial working memory was significantly related to perspective taking and empathetic concern. As an individual’s visual-spatial working memory decreased, cognitive empathy and affective empathy significantly decreased as well, indirectly increasing the amount of aggression men displayed during the conflict discussion. This is consistent with our current theoretical rationale that empathetic processes mediate the relation between executive functioning and intimate partner aggression observed in the lab. A similar pattern was found for frequency of physical abuse outside of the lab. The negative relation between working memory and physical IPV was fully mediated through affective empathy, but not cognitive empathy. Violence inhibition appears to be linked more strongly to affective processes and empathic concern rather than the ability to cognitively take another’s perspective.

While the constructs of working memory and empathy appear to be quite distinct, empathy appears to be dependent upon the ability to decode complex, emotional information from other humans and hold that information in long enough to formulate an appropriate response. Sex offender researchers posit four main components comprising the stages of an empathic response: (a) emotional recognition, (b) perspective taking, (c) emotional replication, and (d) response decision, e.g., a decision not to use violence [43]. Deficits in working memory can interfere with the latter three stages in generating an empathic, non-aggressive response.

Within the study, there were no differences between men with and without a history of head trauma on working memory or empathy within our sample. This is most likely due to our small sample size, with only 14 men reporting a history of concussions. However, this is also possibly due to the phrasing on the questionnaire regarding lack of detail that was presented to the participants as what met a requirement for a concussion. Future studies should examine these mechanisms with questionnaires that have been empirically supported to reliably and validly assess for concussions among participants.

The lack of significant findings between individuals with no head injury history and those who reported concussions does raise the question as to what caused individuals to have deceased working memory within our sample. We speculate that deficits in working memory that decreased empathetic functioning could have potentially been caused by a number of other factors that influence neurological functioning that we failed to consider, such as past incidents of hypoxia, illnesses, or congenital predispositions. Thus, our single concussion question item ultimately failed to capture the proper range of head injuries or other factors that might have influenced working memory.

There were a few limitations to the present study. First, due to the nature of the cross-sectional data analysis, we cannot make causal conclusions. Therefore, causation cannot be clearly established between empathy, deficits in visual-spatial processing and working memory, and aggression toward an intimate partner. Second, although the community sample was ethnically diverse, it was homogeneous with respect to sexual orientation. Only heterosexual men were included in the analyses. Given that there are gender-related differences in visual-spatial processing and empathy, [44,45], results may not generalize to female perpetrators or to homosexual relationships or to court-mandated or shelter samples. Another limitation is that the 15-min laboratory-based conflict discussion task may not realistically capture conflict in the private; because participants know they are being recorded, it is possible they may behave more appropriately during the discussion, i.e., the Hawthorne effect [46]. Future studies would have participants wear in-home portable recording devices so behaviors could be videotaped in a more naturalistic setting. Finally, due to the interdependent nature of empathetic concern and perspective taking, we are unable to determine if there is a more complex mediation process between these factors and working memory. For the models estimating aggression observed during the conflict, both factors fully mediated working memory. Working memory may be associated with just one of these factors that later influences the other in a serial mediation process. Unfortunately, due to our sample size, testing all factors in a single serial mediation was not possible due to limited power.

## 5. Conclusions

Despite these caveats, these findings have important implications for researchers examining etiological models of aggression and violence and for clinicians in both physical and mental health settings. The present study demonstrates that the link between men’s visual-spatial working memory and both observed partner aggression and partners’ reports of his violence through empathy. These effect sizes are large, suggesting a robust and clinically significant relation between neuropsychological functioning, empathy and partner aggression.

Currently, most battering intervention programs focus on changing attitudes and beliefs using Duluth Model or Cognitive Behavioral approaches [47]. Few battering intervention programs incorporate aspects of executive function, empathic concern or perspective taking. There are existing interventions designed to improve short-term working memory [48] and empathy [28]. Addressing empathy and neuropsychological deficits therapeutically may increase the efficacy of battering intervention programs. Tailoring interventions specifically for perpetrators with deficits in working memory may prove fruitful in increasing the efficacy of battering intervention programs.

## Figures and Tables

**Table 1:** Descriptive statistic and bivariate correlations.

		1	2	3	4	5	6
1. Working Memory							
2. Cognitive Empathy		0.44 **					
3. Affective Empathy		0.38 **	0.60 ***				
4. Observed Aggression		−0.30 *	−0.47 ***	−0.46 **			
5. Physical IPV Perpetration		−0.34 **	−0.29 *	−0.38 **	0.23		
6. Psychological IPV Perpetration		−0.22	−0.17	−0.34 **	0.44 **	0.53 ***	
Mean Standard Deviation	Concussion	60.49	16.53	18.13	16.60	3.60	31.53
		5.21	1.13	0.80	5.65	2.62	4.19
Mean	No Concussion	53.32	16.00	17.52	22.44	8.37	23.98
Standard Deviation		3.46	0.75	0.53	3.76	1.74	24.71
*F* Statistic (*p* value)		1.13 (0.26)	0.15 (0.70)	0.41 (0.53)	0.39 (0.14)	2.30 (0.14)	2.07 (0.16)

Note: * p < 0.05, ** *p* < 0.01, *** *p* < 0.001; Working memory (Correct percentile on CBBT [29]); Cognitive Empathy and Affective Empathy (Perspective taking and Empathetic Concern; [36]); Observed Aggression (Sum of Coded aggressive affect Onset, [32], Female partner report of male IPV(Rate of psychological or physical assault perpetrated within the last year [31]).
